# Influence of the *ABCB1-rs1045642* gene polymorphism on blood drug concentration in voriconazole-treated patients with severe invasive fungal infection

**DOI:** 10.3389/fphar.2025.1510890

**Published:** 2025-03-28

**Authors:** Qian Zhang, Xing Gao, Dongmei Lv

**Affiliations:** ^1^ Xuzhou Medical University, Xuzhou, China; ^2^ Second Affiliated Hospital of Xuzhou Medical University, Xuzhou, China; ^3^ Affiliated Hospital of Xuzhou Medical University, Xuzhou, China

**Keywords:** *ABCB1-rs1045642*, gene polymorphism, inheritance, voriconazole, blood concentration, invasive fungal infection

## Abstract

This study aimed to investigate the impact of the *ABCB1-rs1045642* gene polymorphism on the blood drug concentrations of voriconazole in patients with severe invasive fungal infections. A total of 101 patients treated with voriconazole were enrolled in this study. Polymerase chain reaction and Sanger sequencing were used to detect the genotype of *ABCB1-rs1045642*, and enzyme amplified immunoassay was used to detect the plasma trough concentration of voriconazole. We analyzed the impacts of patient genotype and the minimum concentration of voriconazole as well as investigated the treatment efficacy and rates of adverse reactions in patients with different genotypes. All subjects received standard-dose voriconazole treatment for 1 week, and the mean plasma concentration was found to be 4.5 (3.10, 6.90) mg/L. Three genotypes of *ABCB1-rs1045642* were found in the study cohort, namely, wild type (CC type), heterozygous mutant type (CT type), and homozygous mutant type (TT type). There were 18 TT, 48 CT, and 35 CC type cases. Patients with different genotype groups and varying plasma trough concentrations did not differ statistically significantly in terms of the treatment efficacy or incidence of adverse events. Voriconazole plasma concentrations differed significantly among patients of different genders and *ABCB1-rs1045642* genotypes. By incorporating gender into the multiple regression model, the regression equations were obtained as C1 = 6.09–1.33×Gender (male = 0, female = 1)-0.47× X1 (X1: T/T = 1, non-T/T = 0) and C2 = 6.09–1.33×Gender (male = 0, female = 1)-0.94×X1 (X1: T/T = 1, non-T/T = 0). The *ABCB1-rs1045642* genotype was not found to affect voriconazole plasma trough concentrations in patients with invasive fungal infections admitted to the intensive care unit.

## 1 Introduction

The mortality rates of invasive fungal infections (IFIs) in patients in the intensive care unit (ICU) are quite high and second only to those of patients with hematological cancers. ICU patients are particularly vulnerable to IFIs (Enoch et al., 2017). At present, the most commonly used antifungal drug in clinical practice is triazole voriconazole, but it was found that there were large differences in the therapeutic effects on different patients during its use. Different factors, such as age, gender, body mass index (BMI), *CYP2C19* gene polymorphism, liver disease, inflammation, and drug interactions, could contribute to this interindividual variability (Allegra et al., 2018). The same dose of treatment has been reported to produce uneven therapeutic effects, and some patients may even have adverse reactions of varying severity (Schulz et al., 2019). When voriconazole is administered to an adult at doses exceeding 3 mg/kg q12h by intravenous infusion, it exhibits non-linear pharmacokinetic properties *in vivo* (Purkins et al., 2002; Rosanova et al., 2018). Significant variations in drug plasma exposure have been observed among patients (Allegra et al., 2018).

Genetically speaking, polymorphisms of the *CYP2C19, CYP2C9*, and *CYP3A4* genes are known to significantly influence the metabolism of voriconazole (Owusu Obeng et al., 2014). Among these, the loss-of-function alleles of *CYP2C19*, such as *2 and *3, can retard metabolism and increase the blood drug concentration. This is particularly evident in Asian populations; owing to the high frequencies of these loss-of-function alleles in Asian populations, their plasma drug concentrations are more likely to be elevated. The single-nucleotide polymorphism (SNP) of the transporter gene *ABCB1-rs1045642* can alter the functions of P-glycoproteins (P-gps) to affect the absorption and distribution of the drug, thereby changing the blood drug concentration. These genetic characteristics are of great significance for individualized uses of voriconazole and provide a basis for precise clinical medication (Zaini et al., 2020).

Recent studies have shown that SNPs of the *ABCB1-rs1045642* gene can impact P-gp functions or activities, which are closely linked to variations in the *in vivo* responses of different drugs (Cascorbi, 2011; Pramanik et al., 2014). The *ABCB1-rs1045642* gene polymorphisms are associated with voriconazole pharmacokinetics (Weiss et al., 2009; Allegra et al., 2018), whereby the voriconazole trough concentrations and *ABCB1-rs1045642* SNPs are linked (Allegra et al., 2018). The *ABCB1* gene polymorphism decreases the P-gp function, which in turn is associated with an increase in the plasma concentration of voriconazole by approximately 15%–20% (Brossard et al., 2014).

The main purpose of this study was to determine whether the *ABCB1-rs1045642* gene polymorphism is associated with the blood drug concentrations of voriconazole in patients with severe IFIs. By understanding this relationship, we aim to provide a scientific basis for personalized dosing of voriconazole, which could improve the therapeutic efficacy and safety of voriconazole treatment in these patients.

## 2 Patients and methods

### 2.1 Patients and inclusion/exclusion criteria

A total of 101 patients were included in this study; these participants were diagnosed with an IFI between January 2023 and June 2024 by the departments of emergency critical care medicine and respiratory critical care medicine of the affiliated hospital of Xuzhou Medical University. *The Guidelines for the Diagnosis and Treatment of Invasive Fungal Infection in Critically Ill Patients* (Von Lilienfeld-Toal et al., 2019) was used as the foundation for the diagnostic criteria. The patient inclusion requirements are as follows:

(1) Patient should fulfill the necessary diagnostic requirements for an IFI.
(2) Patient must be at least 18 years old.
(3) Patient should possess comprehensive clinical data.

Some exclusion criteria were also used on the study group members as follows:

(1) Severe hepatic or renal impairment.
(2) Failure to obtain a sample of plasma drug concentration.
(3) Concurrent or ongoing use of other antifungal medications.
(4) Contraindications to voriconazole.
(5) Use of medications that interfere with voriconazole metabolism.
(6) Nursing or pregnant patients.

The voriconazole treatment regimen was as follows: a loading dose of 6 mg/kg q12h was administered initially as either intravenous drip or nasogastric administration; then, the maintenance dose of 4 mg/kg q12h was administered starting from the second day (Jiang and Lin, 2024). This study was approved by the ethics committee of the Affiliated Hospital of Xuzhou Medical University (approval number: XYFY2023-KL481-01).

### 2.2 Determinations of voriconazole plasma concentration

#### 2.2.1 Testing instrument

The fully automatic biochemical analyzer from Alytech Group (model: Vivo-ProE) was used as the testing instrument in this study.

#### 2.2.2 Plasma drug concentration determination

In this study, the voriconazole dosage was adjusted using prespecified algorithms based on voriconazole trough concentrations in the target range of 1–5.5 mg/mL (Pasqualotto et al., 2010), and the linear range of voriconazole was 0.5–16 μg/mL. The detection limit was 0.5 μg/mL. On the morning of the third day of medication, the medical staff collected 2 mL of fasting venous anticoagulated whole blood from each patient; then, 4 µL of the processed blood sample was used to perform enzyme amplification immunoassay, and the fully automatic biochemical analyzer at the Gene Testing Laboratory of Alytech Group was used to monitor the patient’s blood drug concentration. Glucose 6-phosphate dehydrogenase (G6PDH) was labeled on the voriconazole molecule by enzyme amplified immunoassay. During measurement, voriconazole and G6PDH*-*labeled voriconazole were quantified in the samples bound to the anti-voriconazole antibody in the reagent through immunocompetition; however, the binding of the latter to the antibody resulted in a decrease in the activity of G6PDH. Thus, when voriconazole is present in the sample, the activity of G6PDH increases relatively, thereby converting the oxidized nicotinamide adenine dinucleotide (NAD^+^) in the reagent to reduced nicotinamide adenine dinucleotide (NADH), which has a specific absorption peak at 340 nm. The rate of change of NADH absorbance at this wavelength is proportional to the concentration of voriconazole in the sample, which then allows calculation of the concentration of voriconazole in the sample (Osipenko and Garkushina, 2022).

### 2.3 Genotype detection

#### 2.3.1 Detection instrument

The polymerase chain reaction (PCR) amplifier (ABI 3730, Applied Biosystems, United States) was used as the detection instrument in this study.

#### 2.3.2 Genotyping procedure

The remaining venous blood from each draw was used to extract the sample DNA with the Tiangen Blood Genomic DNA Extraction Kit (batch no. 20220321) based on manufacturer instructions. The amplification primers for the *ABCB1-rs1045642* gene were synthesized by Shanghai Shenggong Biotechnology Co., Ltd., and the amplification was achieved through PCR on the DNA samples. The primer sequences are as follows:

upstream primer: 5′-TGT​TTT​CAG​CTG​CTT​GAT​GG-3′
downstream primer: 5′-AAG​GCA​TGT​ATG​TTG​GCC​TC-3′
sequencing primer: 5′-GGC​CTC​CTT​TGC​TGC-3′

Shanghai Shenggong Company also assisted with performing the gene sequencing and PCR amplification using capillary electrophoresis and four-color fluorescent dye-labeled dideoxynucleoside triphosphates (ddNTPs) (terminator method). Here, a single-primer PCR sequencing was used to generate a single-stranded DNA mixture labeled with four distinct fluorescent dyes, where each differed by one base at the 3′ end. These PCR products were then subjected to capillary electrophoresis. Owing to their varying molecular sizes, their migration rates differed. As the DNA mixture passed through the capillary reading window, a charge-coupled device (CCD) camera detector captured the fluorescent signals sequentially. A grating was used to separate the emitted fluorescence based on color, where each color represented a specific base. The imaging process was synchronized with the CCD camera, and the analysis software automatically converted the fluorescence signals into DNA sequences to complete the sequencing. Based on the findings of the gene sequencing, the *ABCB1-rs1045642* genotypes were classified into three categories as CC, CT, and TT types.

### 2.4 Voriconazole treatment efficacy and adverse reaction incidence

Data were collected from the patients, including their gender, age, BMI, diagnosis at admission, outcome, liver function, renal function, infection index, fungal G/GM tests, albumin level, as well as Acute Physiology and Chronic Health Evaluation II (APACHE-II) score. The adverse reactions of voriconazole include liver function damage, neurotoxicity, visual impairment (e.g., decreased visual acuity and blurred vision), skin reactions, and reactions related to intravenous infusion (e.g., phlebitis and pain at the infusion site). The efficacy of voriconazole treatment was determined according to the *Guidelines for the Diagnosis and Treatment of Invasive Fungal Infections in Critically Ill Patients* (Von Lilienfeld-Toal et al., 2019).

### 2.5 Statistical analysis

The tool used for data analysis was SPSS 27.0. The non-normal distribution data were expressed as median and interquartile range (IQR) [M (P25–P75)], and non-parametric tests were used for the intergroup comparisons. The count data were expressed as cases (%), and the normally distributed measurement data were expressed as $\bar{x}$ *± s,* where t-tests were used for the intergroup comparisons. In the two-tailed test, a *p*-value of less than 0.05 indicated a statistically significant difference. The chi-squared (χ^*2*^) test was employed to assess the efficacy of voriconazole treatment and occurrence rate of its adverse reactions in patients with diverse genotypes. Multiple linear regression was utilized to analyze the factors influencing the minimum concentration (C_min_) of voriconazole.

## 3 Results

### 3.1 General information about the patients

A total of 101 patients with IFIs who were treated with voriconazole were enrolled in this study. The demographic, clinical, and pharmacokinetic features of our cohort are summarized in Table 1.

**TABLE 1:** Demographic, clinical, and pharmacokinetic characteristics of the enrolled patients.

Variable	n = 101
Gender	
Male, n (%)	70 (69.3)
Female, n (%)	31 (30.7)
Age (years)	
Median (IQR)	66.7 (55–76)
BMI (kg/m^2^)	
Median (IQR)	23.30 (20.65–24.88)
Albumin level (g/L)	
Median (IQR)	30.31 (26.9–33.9)
Route of administration	
Intravenous infusion, n (%)	81 (80.2)
Nasogastric administration, n (%)	20 (19.8)
Drug dose (mg/kg)	
Median (IQR)	6.47 (5.35–8.00)
Serum creatinine (µmol/L)	
Median (IQR)	83.12 (33.75–101.25)
Voriconazole concentration (μg/mL)	
Median (IQR)	5.28 (2.83–6.97)
APACHE-II Score	
Median (IQR)	19.00 (13.00–24.75)

IQR, Interquartile range; n, Number; APACHE-II, Acute Physiology and Chronic Health Evaluation II; BMI, body mass index.

### 3.2 *ABCB1-rs1045642* genotype results

**FIGURE 1:**
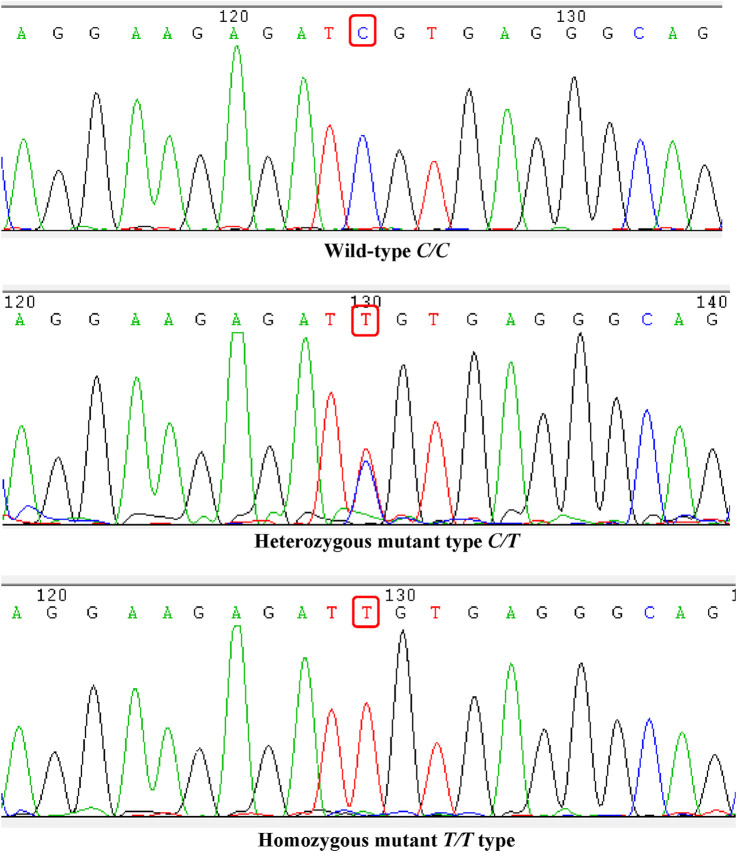
Genotype sequencing of the *ABCB1-rs1045642* locus.

All patients in the cohort were genotyped for *ABCB1-rs1045642*, which showed that there were 35 CC types (wild type, where C represents the wild allele), 48 CT types (heterozygous mutation, where C represents the wild allele and T represents the mutant allele), and 18 TT types (homozygous mutation, where T represents the mutant allele). The sequencing results are shown in Figure 1. The genotypes of the study samples are in line with the Hardy–Weinberg equilibrium (χ^*2*^ = 0.048, *p* > 0.05), which suggests that the patients chosen are representative and suitable for further research (Figure 1).

### 3.3 Voriconazole C_min_ comparisons in the patients

**FIGURE 2:**
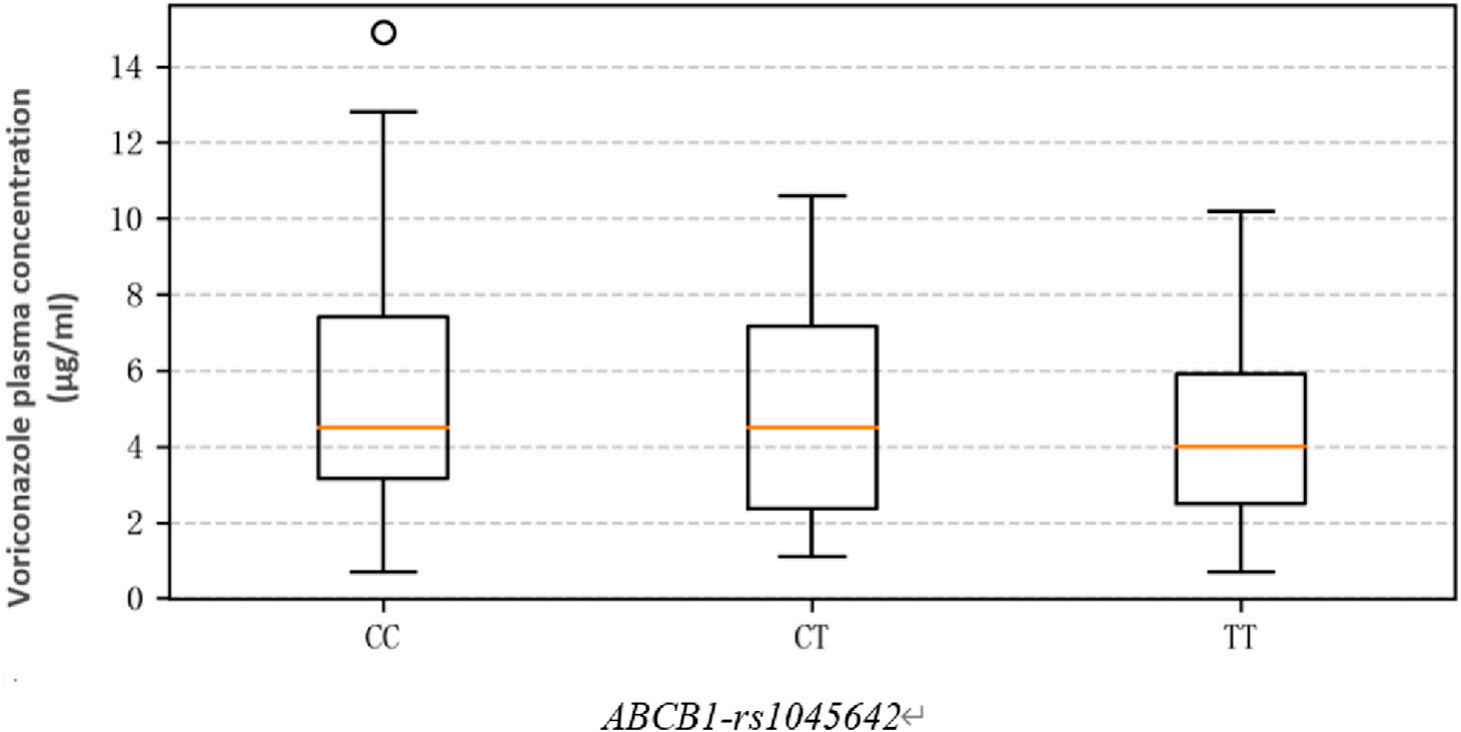
Influence of *ABCB1-rs1045642* SNPs on voriconazole plasma trough concentrations.

The average C_min_ of the study cohort was 4.5 (3.10, 6.90) mg/L, and the linear range was 0.5–16 mg/L. The voriconazole plasma trough concentrations (mg/L) in patients with different genotypes of *ABCB1-rs1045642* were as follows: CC 4.50 (3.15, 7.40); CT 4.50 (3.00, 6.93); TT 4.00 (2.67, 6.20). The results show that the C_min_ values in the CC group are higher than those in the CT and TT groups, but there is no significant difference (χ^*2*^ = 0.095, *p* = 0.623). Thus, genotype differences were not found to have significant effects on the plasma trough concentrations (Figure 2).

Based on data processing and graphical analysis, the plasma trough concentrations of voriconazole among the patients with CC, CT, and TT genotypes did not exhibit significant differences in either the degree of dispersion or central tendency (Li et al., 2024). This indicates that the different genotypes may have relatively slight impacts on the plasma drug concentration, which is consistent with the conclusion of this study.

### 3.4 Effectiveness and adverse reaction rate of voriconazole

This section presents a comparison of the effectiveness and adverse reaction rates of voriconazole in patients with different *ABCB1-rs1045642* genotypes.

#### 3.4.1 Overall efficacy and adverse reactions of all patients

Of the 101 patients treated in this study, the overall efficacy of voriconazole was 70.30% (71/101); abnormal liver functions were observed in 12 patients and rashes were observed in two patients.

#### 3.4.2 Efficacy and adverse reaction rates by genotype

CC Type: Efficacy was 62.86% (22/35) and adverse reaction rate was 8.57% (3/35).

CT Type: Efficacy was 77.08% (37/48) and adverse reaction rate was 18.75% (9/48).

TT Type: Efficacy was 66.67% (12/18) and adverse reaction rate was 11.11% (2/18).

Comparatively, the CT genotype showed relatively higher efficacy than the CC and TT genotypes, while the adverse reaction rates varied among the three genotypes without significant overall differences.

#### 3.4.3 Statistical significance

Statistically, there were no significant differences in the effectiveness and adverse reaction rates for voriconazole treatment among the three genotypes (*p* > 0.05), as shown in Table 2.

**TABLE 2:** Comparison of treatment effects and adverse reactions in patients with different *ABCB1-rs1045642* genotype cases (%).

Genotype	n	Treatment efficiency	Incidence of adverse reactions
C/C	35	22 (62.86)	3 (8.57)
C/T	48	37 (77.08)	9 (18.75)
T/T	18	12 (66.67)	2 (11.11)
*χ* ^2^		2.10	1.89
*p*		0.411	0.388

### 3.5 Comparison of therapeutic efficacy and adverse reaction rates for different voriconazole trough concentrations

According to the individualized medication guide for voriconazole (Pasqualotto et al., 2010), the voriconazole blood concentration was divided into three groups as C_min_ <1.0 mg/L, 1.0 ≤ C_min_ ≤ 5.5 mg/L, and C_min_ >5.5 mg/L. Among the three groups with varying C_min_, there were no statistically significant variations in the occurrence of adverse reactions or treatment effectiveness (*p* > 0.05), as shown in Table 3.

**TABLE 3:** Comparison of therapeutic effects and adverse reactions in patients with different voriconazole C_min_ cases (%).

Voriconazole C_min_ (mg/L)	n	Effective rate of drug treatment	Incidence rate of adverse reactions
C_min_ <1.0	4	3 (75.00)	0 (0.00)
1.0 ≤ C_min_ ≤ 5.5	58	39 (67.24)	10 (16.24)
C_min_ >5.5	39	29 (74.36)	4 (10.25)
*p*		0.792	0.582

### 3.6 Impacts of clinically relevant non-genetic variables on voriconazole C_min_

The rank-sum test was conducted on the C_min_ levels of each group after the patients were categorized based on factors such as age, gender, BMI, underlying diseases, method of administration, albumin level, and APACHE-II scores. Table 4 indicates that there was a significant difference (*p* < 0.05) in the voriconazole C_min_ values between the male and female patient groups; this suggests that gender may be an important factor affecting voriconazole C_min._ Further analyses of the other factors and their relationships with voriconazole C_min_ are also of great significance and will be discussed in the following sections.

**TABLE 4:** Effects of non-genetic factors on voriconazole C_min_.

Classification	C_min_ (mg/L) *[M (P25, P75)]*	*Z*	*P*
Gender			
Male	4.90 (3.15, 7.30)	2.03*	0.042*
Female	3.7 (2.95, 4.85)		
Age (years)			
<60	4.10 (2.65, 7.10)	0.73	0.648
≥60	4.60 (3.10, 6.60)		
Albumin level (g/L)			
<35	4.50 (3.10, 6.90)	1.47	0.141
≥35	1.20 (1.20, 1.20)		
BMI (kg/m^2^)			
<24	4.00 (3.10, 5.90)	1.03	0.141
≥24	4.55 (3.50, 6.67)		
Route of administration			
Intravenous drip	4.50 (3.20, 7.20)	1.66	0.096
Nasogastric administration	3.20 (2.60, 6.15)		
Basic diseases			
Including	4.70 (3.30, 7.05)	1.32	0.188
Excluding	4.20 (2.65, 6.75)		
APACHE-II			
<15	3.55 (2.15, 5.02)	1.82	0.354
≥15	4.50 (3.80, 6.60)		

M *(P25, P75),* median (25th percentile, 75th percentile); *Z, Z*-score; APACHE-II, Acute Physiology and Chronic Health Evaluation II; BMI, body mass index.

### 3.7 Multiple linear regression analysis of factors influencing voriconazole C_min_

Since multiple variables (such as gender and genotype) are involved in this study and included in the model for testing, the Bonferroni correction was adopted to control the error rates of multiple tests before the multiple linear regression analysis. In this step, the overall significance level was set to *α* = 0.05. The regression analysis involved testing of three variables, namely, gender, C/T, and T/T. After Bonferroni correction, the significance level for each variable was adjusted to *α'* = 0.05/3 = 0.017.

When interpreting the findings of the regression analysis, *α′* is used as the criterion for determining statistical significance. After obtaining the results of the regression analysis, the *p*-value of the t-test of each variable was compared with *α'* = 0.017. A variable is considered statistically significant at the adjusted significance level when its *p*-value is less than 0.017. Conversely, if the *p*-value is 0.017 or greater, the variable is not statistically significant at this adjusted significance level.

Gender was found to be a statistically significant variable; furthermore, the genotypes of A*BCB1-rs1045642* were included in the multiple linear regression analysis model to obtain the following equations:

$$C1=6.09\;–1.33\times \text{Gender }\left(\text{male}=0,\text{female}=1\right)-0.47\times X1\;\left(X1:C/T=1,\text{non}-C/T=0\right);$$

$$C2=6.09\;–1.33\times \text{Gender }\left(\text{male}=0,\text{female}=1\right)-0.94\times X1\;\left(X1:T/T=1,\text{non}-T/T=0\right).$$

After controlling for the impact of gender on voriconazole C_min_, as indicated in Table 5, the genotypes of the *ABCB1-rs1045642* gene did not significantly affect C_min_ *(R* ^*2*^ = 0.038, *p* = 0.152).

**TABLE 5:** Multiple linear regression analysis of factors affecting voriconazole C_min_.

Project	*β*	*S.E*	*t*	*p*
Gender Female	−1.33	0.74	−1.81	0.074
*C/T*	−0.47	0.76	−0.62	0.539
*T/T*	−0.94	0.99	−0.95	0.345

β, Beta coefficient; S.E, standard error; t, t-statistic; *p*, probability.

## 4 Discussion

The present study investigates the influence of the *ABCB1-rs1045642* gene polymorphism on voriconazole trough concentrations in patients with severe IFIs. Although significant associations were not found between the *ABCB1-rs1045642* polymorphisms and voriconazole concentrations, a notable finding was the significantly higher voriconazole trough levels in male patients compared to females. This observation highlights the potential role of sex as a critical factor in voriconazole pharmacokinetics and warrants further discussion.

### 4.1 Gender differences in voriconazole pharmacokinetics

Several factors may contribute to the gender difference phenomenon:

(1) Physiological differences

Men generally have higher body weights and lean body masses compared to women, which can influence the volume of distribution (Vd) of drugs. Voriconazole is a lipophilic drug and may be distributed differently in males owing to variations in the body composition. Sex-specific differences in hepatic blood flow and enzyme activity, particularly cytochrome P450 (*CYP*) enzymes such as *CYP2C19* that is primarily responsible for voriconazole metabolism, may contribute to the observed disparity. Studies have shown that *CYP2C19* activity can vary between the sexes, potentially leading to differences in drug clearance (Allegra et al., 2020).

(2) Hormonal influences

Sex hormones, such as estrogen and testosterone, have been reported to modulate the expressions and activities of drug-metabolizing enzymes and transporters. For instance, estrogen has been shown to downregulate *CYP3A4* activity, which could indirectly affect voriconazole metabolism. The interplay between hormonal fluctuations and drug pharmacokinetics is particularly relevant in the case of premenopausal women, who were likely included in our study population.

(3) Drug transporters

Although the *ABCB1-rs1045642* polymorphisms did not show significant impacts in our study, sex-based differences in the expressions and functions of drug transporters, including P-gp that is encoded by the *ABCB1* gene (Nazir et al., 2020), cannot be ruled out. Previous studies have suggested that males may have higher P-gp activities, which could potentially influence drug distribution and elimination.

### 4.2 Clinical implications

The higher voriconazole trough concentrations in men may have important clinical implications. Voriconazole exhibits a narrow therapeutic index (Moriyama et al., 2015), and elevated concentrations of the drug are associated with increased risks of adverse effects, such as hepatotoxicity and neurotoxicity. Therefore, our findings suggest that the sex of the patient should be considered when optimizing their voriconazole dosing regimen. For instance, male patients may require lower initial doses or more frequent therapeutic drug monitoring to avoid toxicity; conversely, female patients may need higher doses to achieve therapeutic concentrations, particularly in cases of treatment failure or suboptimal responses.

### 4.3 Limitations

Several limitations of this study should be acknowledged. First, the sample size was relatively small, which may have limited the power to detect significant associations, particularly with regard to genetic polymorphisms. Second, we did not account for other potential confounding factors, such as dietary habits, concomitant medications, or hormonal status, which could influence voriconazole pharmacokinetics. Third, the study population was restricted to patients with severe IFIs, which could limit the generalizability of our findings to other patient populations.

### 4.4 Future directions

Future studies should aim to validate our findings in larger and more diverse cohorts. Additionally, mechanistic studies are needed to elucidate the underlying causes of sex-based differences in voriconazole pharmacokinetics. For example, we recommend investigating the impacts of sex hormones on *CYP2C19* and *ABCB1* activities as well as exploring the roles of other genetic variants and their interactions with sex-based differences. We also suggest conducting population pharmacokinetic modeling to develop sex-specific dosing algorithms.

## 5 Conclusion

Our current research emphasizes the vital role of considering patient sex as a critical factor in the pharmacokinetics of voriconazole. Although the *ABCB1-rs1045642* polymorphisms did not significantly influence voriconazole concentrations, the observed sex-based differences underscore the need for personalized dosing strategies to optimize the therapeutic outcomes while minimizing adverse effects. Therefore, additional research efforts are needed to comprehensively understand the mechanisms behind these differences and apply the findings to clinical practice.

## Data Availability

The dataset presented in this study can be found in online repositories. The name of the repository and accession number can be found below: http://www.ncbi.nlm.nih.gov/gene/5243.

## References

[B1] Allegra S. De Francia S. De Nicolò A. Cusato J. Avataneo V. Manca A. (2020). Effect of gender and age on Voriconazole Trough concentrations in Italian adult patients. Eur. J. Drug Metab. Pharmacokinet. 45, 405–412. 10.1007/s13318-019-00603-6 31965553

[B2] Allegra S. Fatiguso G. Francia S. D. Pirro E. Carcieri C. Cusato J. (2018). Pharmacogenetic of voriconazole antifungal agent in pediatric patients. Pharmacogenomics 19, 913–925. 10.2217/pgs-2017-0173 29914286

[B3] Brossard P. Scherz M. Halabi A. Maatouk H. Krause A. Dingemanse J. (2014). Multiple-dose tolerability, pharmacokinetics, and pharmacodynamics of ponesimod, an S1P_1_ receptor modulator: favorable impact of dose up-titration: the Journal of Clinical Pharmacology. J. Clin. Pharmacol. 54, 179–188. 10.1002/jcph.244 24408162

[B4] Cascorbi I. (2011). “P-Glycoprotein: tissue distribution, substrates, and functional consequences of genetic variations,” in Drug transporters. Editors Fromm M. F. Kim R. B. (Berlin, Heidelberg: Springer Berlin Heidelberg), 261–283. 10.1007/978-3-642-14541-4_6 21103972

[B5] Enoch D. A. Yang H. Aliyu S. H. Micallef C. (2017). “The changing epidemiology of invasive fungal infections,” in Human fungal pathogen identification. Editor Lion T. (New York, NY: Springer New York), 17–65. 10.1007/978-1-4939-6515-1_2 27837497

[B6] Jiang L. Lin Z. (2024). Voriconazole: a review of adjustment programs guided by therapeutic drug monitoring. Front. Pharmacol. 15, 1439586. 10.3389/fphar.2024.1439586 39712496 PMC11658975

[B7] Li X. Hu Q. Xu T. (2024). Associated factors with voriconazole plasma concentration: a systematic review and meta-analysis. Front. Pharmacol. 15, 1368274. 10.3389/fphar.2024.1368274 39246651 PMC11377273

[B8] Moriyama B. Kadri S. Henning S. A. Danner R. L. Walsh T. J. Penzak S. R. (2015). Therapeutic drug monitoring and genotypic screening in the clinical use of voriconazole. Curr. Fungal Infect. Rep. 9, 74–87. 10.1007/s12281-015-0219-0 26918067 PMC4764088

[B9] Nazir S. Adnan K. Gul R. Ali G. Saleha S. Khan A. (2020). The effect of gender and ABCB1 gene polymorphism on the pharmacokinetics of azithromycin in healthy male and female Pakistani subjects. Can. J. Physiol. Pharmacol. 98, 506–510. 10.1139/cjpp-2019-0569 32125889

[B10] Osipenko A. Garkushina I. (2022). The effect of the synthesis method on physicochemical properties of selective granular polymer sorbents. Polymers 14, 353. 10.3390/polym14020353 35054763 PMC8778989

[B11] Owusu Obeng A. Egelund E. F. Alsultan A. Peloquin C. A. Johnson J. A. (2014). *CYP 2C19* polymorphisms and therapeutic drug monitoring of voriconazole: are we ready for clinical implementation of pharmacogenomics? Pharmacotherapy 34, 703–718. 10.1002/phar.1400 24510446 PMC4082739

[B12] Pasqualotto A. C. Xavier M. O. Andreolla H. F. Linden R. (2010). Voriconazole therapeutic drug monitoring: focus on safety. Expert Opin. Drug Saf. 9, 125–137. 10.1517/14740330903485637 20021293

[B13] Pramanik S. Surendran S. T. Devi S. Krishnamurthi K. Chakrabarti T. (2014). Frequency and genotype distribution of *ABCB1* gene polymorphisms among Maharashtra population of Central India. Xenobiotica 44, 579–582. 10.3109/00498254.2013.866300 24308438

[B14] Purkins L. Wood N. Ghahramani P. Greenhalgh K. Allen M. J. Kleinermans D. (2002). Pharmacokinetics and safety of voriconazole following intravenous-to oral-dose escalation regimens. Antimicrob. Agents Chemother. 46, 2546–2553. 10.1128/AAC.46.8.2546-2553.2002 12121931 PMC127341

[B15] Rosanova M. T. Bes D. Serrano Aguilar P. Sberna N. Lede R. (2018). Efficacy and safety of voriconazole in immunocompromised patients: systematic review and meta-analysis. Infect. Dis. 50, 489–494. 10.1080/23744235.2017.1418531 29262742

[B16] Schulz J. Kluwe F. Mikus G. Michelet R. Kloft C. (2019). Novel insights into the complex pharmacokinetics of voriconazole: a review of its metabolism. Drug Metab. Rev. 51, 247–265. 10.1080/03602532.2019.1632888 31215810

[B17] Von Lilienfeld-Toal M. Wagener J. Einsele H. Cornely O. A. Kurzai O. (2019). Invasive fungal infection. Dtsch. Ärzteblatt Int. 10.3238/arztebl.2019.0271 PMC6549129 31159914

[B18] Weiss J. Ten Hoevel M. M. Burhenne J. Walter‐Sack I. Hoffmann M. M. Rengelshausen J. (2009). *CYP2C19* genotype is a major factor contributing to the highly variable pharmacokinetics of voriconazole. J. Clin. Pharma 49, 196–204. 10.1177/0091270008327537 19033450

[B19] Zaini F. Lotfali E. Fattahi A. Siddig E. Farahyar S. Kouhsari E. (2020). Voriconazole resistance genes in Aspergillus flavus clinical isolates. J. de Mycol. Médicale 30, 100953. 10.1016/j.mycmed.2020.100953 32362445

